# Polarized localization and borate-dependent degradation of the *Arabidopsis* borate transporter BOR1 in tobacco BY-2 cells

**DOI:** 10.12688/f1000research.2-185.v1

**Published:** 2013-09-13

**Authors:** Noboru Yamauchi, Tadashi Gosho, Satoru Asatuma, Kiminori Toyooka, Toru Fujiwara, Ken Matsuoka

**Affiliations:** 1 Graduate School of Bioresource and Bioenvironmental Sciences, Kyushu University, Fukuoka, 812-8581, Japan; 2 Biotechnology Research Center, The University of Tokyo, Tokyo, 113-8657, Japan; 3 Laboratory of Plant Nutrition, Faculty of Agriculutre, Kyushu University, Fukuoka, 812-8581, Japan; 4 Current address: Omu Milk Products Co., Ltd., Omuta, 836-0895, Japan; 5 RIKEN Plant Science Center, Yokohama, 230-0045, Japan; 6 Current address: RIKEN Center for Sustainable Resource Science, Yokohama, 230-0045, Japan; 7 Current address: Laboratory of Plant Nutrition and Fertilizer, Graduate School of Agricultural and Life Science, The University of Tokyo, Tokyo, 113-8657, Japan; 8 Organelle Homeostasis Research Center, Kyushu University, Fukuoka, 812-8581, Japan; 9 Biotron Application Center, Kyushu University, Fukuoka, 812-8581, Japan

## Abstract

In *Arabidopsis* the borate transporter BOR1, which is located in the plasma membrane, is degraded in the presence of excess boron by an endocytosis-mediated mechanism. A similar mechanism was suggested in rice as excess boron decreased rice borate transporter levels, although in this case whether the decrease was dependent on an increase in degradation or a decrease in protein synthesis was not elucidated. To address whether the borate-dependent degradation mechanism is conserved among plant cells, we analyzed the fate of GFP-tagged BOR1 (BOR1-GFP) in transformed tobacco BY-2 cells. Cells expressing BOR1-GFP displayed GFP fluorescence at the plasma membrane, especially at the membrane between two attached cells. The plasma membrane signal was abolished when cells were incubated in medium with a high concentration of borate (3 to 5 mM). This decrease in BOR1-GFP signal was mediated by a specific degradation of the protein after internalization by endocytosis from the plasma membrane. Pharmacological analysis indicated that the decrease in BOR1-GFP largely depends on the increase in degradation rate and that the degradation was mediated by a tyrosine-motif and the actin cytoskeleton. Tyr mutants of BOR1-GFP, which has been shown to inhibit borate-dependent degradation in *Arabidopsis* root cells, did not show borate-dependent endocytosis in tobacco BY-2 cells. These findings indicate that the borate-dependent degradation machinery of the borate transporter is conserved among plant species.

## Introduction

Boron is one of the essential nutrients for plants, and boron deficiency is a major cause of reduced crop production ^1^. Large quantities of boron are toxic to plants, and boron toxicity is a worldwide problem in food production ^2^. Two different classes of borate transporters were discovered in *Arabidopsis thaliana* ^3^. One of them, a transporter named BOR1, is a plasma membrane borate exporter in *Arabidopsis* root cells, and is essential for efficient xylem loading of boron ^4^. BOR1 and its paralogs are also involved in boron toxicity tolerance in plants ^5, 6^.

It was reported that the level of BOR1 is tightly regulated by the concentration of borate in the growth environment ^7^. At low concentrations of borate, BOR1 is stably localized to the proximal side of plasma membrane in root cells but is degraded upon application of high concentrations of borate ^7– 9^. This degradation occurred after endocytosis of the transporter from the plasma membrane and the endocytosed transporter was transported from early endosome to multivesicular body, ubiquitinated and finally targeted to vacuoles for degradation ^8– 11^. A similar boron-dependent decrease in borate transporter levels was also observed in rice ^12^ although in this case the mechanism of the reduction was not elucidated. Thus it was not clarified whether the borate-induced endocytotic degradation of BOR1 that is found in *Arabidopsis* root cells is conserved among different plant species and different types of plant cells.

The tobacco BY-2 cell line is widely used as a model for the analysis of the cell cycle and protein trafficking in plant cells. This cell line is advantageous for conducting pharmacological studies because of the small size of its cell clumps as well as its ability to grow in liquid suspension ^13^. To obtain an insight into the regulation of borate transporter levels and borate sensing machinery, we investigated the localization and degradation of BOR1-green fluorescent protein (GFP) fusion in tobacco BY-2 cells in the presence of high concentrations of borate and analyzed the effect of inhibitors for protein synthesis, protein degradation and intracellular trafficking on its degradation.

## Results

### Expression of the GFP fusion and *Arabidopsis* borate transporter BOR1 in tobacco BY-2 cells

**Figure 1.**
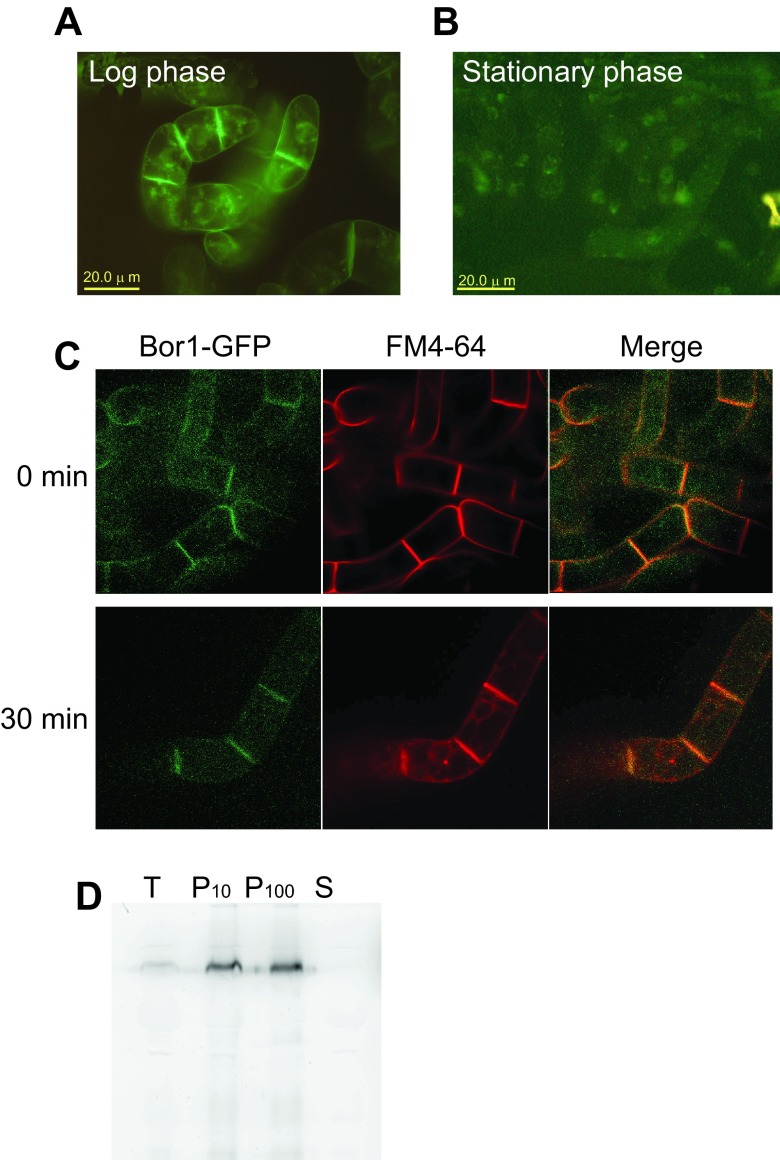
Expression of BOR1-GFP fusion protein in tobacco BY-2 cells. **A**. Fluorescence pattern of rapidly growing tobacco cells expressing BOR1-GFP was detected using an epifluorescence microscope. Plasma membranes as well as some intracellular structures showed GFP fluorescence. **B**. GFP fluorescence of tobacco cells expressing BOR1-GFP at stationary phase of growth. Images were collected as in A. **C**. Colocalization of plasma membrane and endomembrane marker FM4-64 and BOR1-GFP. Cells expressing BOPR1-GFP were suspended in medium containing FM4-64 and incubated for 15 min on ice. Fluorescence images were collected immediately after the incubation or 30 min after incubation at room temperature using a spinning disk confocal microscope. **D**. Membrane-association of BOR1-GFP. Cell lysate was subjected to two rounds of centrifugation and soluble and sedimentable fractions containing membranes was obtained. T, total cell lysate. P10, pellet of 10,000 x g 10 min centrifugation. P100, pellet of 100,000 x g 60 min centrifugation. S, soluble fraction after 100,000 x g 60 min centrifugation.

We expressed the *Arabidopsis* BOR1-GFP fusion construct ^7^ in tobacco BY-2 cells under the control of the cauliflower mosaic virus 35S RNA promoter. Examination of transformed cells at growing phase using an epifluorescence microscope indicated that the fluorescence localized at the most peripheral part of the cells as well as intracellular dots ( Figure 1A). In contrast, cells grown to the stationary-phase showed faint punctate distribution of the fluorescence in the cell ( Figure 1B). To examine whether the peripherally-localized BOR1-GFP in rapidly-growing cells actually localized to the plasma membrane we stained the cells with FM4-64 for 15 min on ice and compared the pattern of FM4-64 and the GFP fluorescence using a spinning disk confocal microscope ( Figure 1C, 0 min). The peripheral staining pattern of FM4-64 is almost completely identical to that of the fluorescence of GFP. When the FM4-64 incubated cells were further incubated at room temperature for 30 min, we observed internalization of the FM4-64 signal. Under this condition we did not observe the internalization of BOR1-GFP signal ( Figure 1C, 30 min). These observations suggest that BOR1-GFP is localized to the plasma membrane in transformed tobacco BY-2 cells.

We also analyzed the distribution of the fluorescence protein after fractionation of the cell lysate into membranous organelle and soluble fractions. As shown in Figure 1D, BOR1-GFP was enriched in the precipitated fractions. This confirmed that BOR1-GFP was targeted to the membranes in tobacco BY-2 cells. Taken together, we concluded that a significant portion of BOR1-GFP expressed in tobacco BY-2 cells is targeted to the plasma membrane as in the case of *Arabidopsis* root cells.

### Borate-dependent decrease of BOR1-GFP in transformed tobacco cells

**Figure 2.**
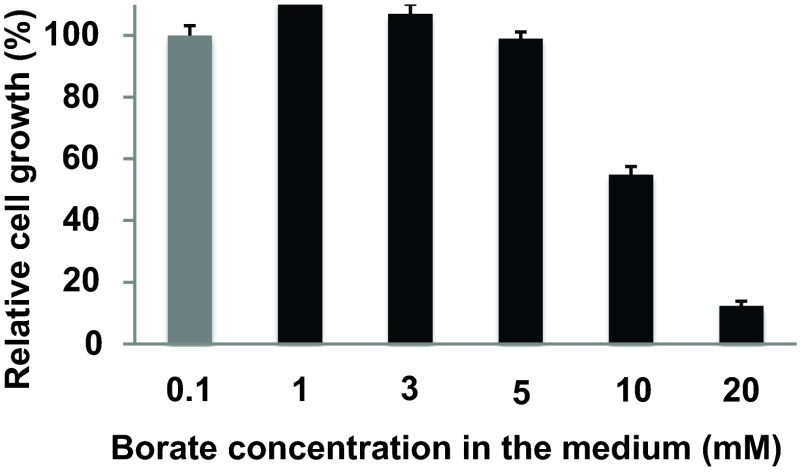
Effect of borate on the growth of BY-2 cells. Seven-day old tobacco BY-2 cells were subcultured into medium containing the indicated concentration of borate and shaken for a week. The volume of cells in the culture was measured. Gray bar; standard borate concentration medium. Error bars, SD.

In order to address whether BOR1-GFP is degraded upon supply of high concentrations of borate in tobacco cells as observed in *Arabidopsis*, we first analyzed the effect of borate on the growth of tobacco cells that expressed the BOR1-GFP. Tobacco cells were grown for a week in medium containing varied concentrations of borate (0.1 to 20 mM) and the volume of cells was measured. No difference in the response to cell growth was observed between non-transformed and BOR1-GFP expressing cells. Medium containing up to 5 mM borate caused little defect in the growth of cells ( Figure 2).

**Figure 3.**
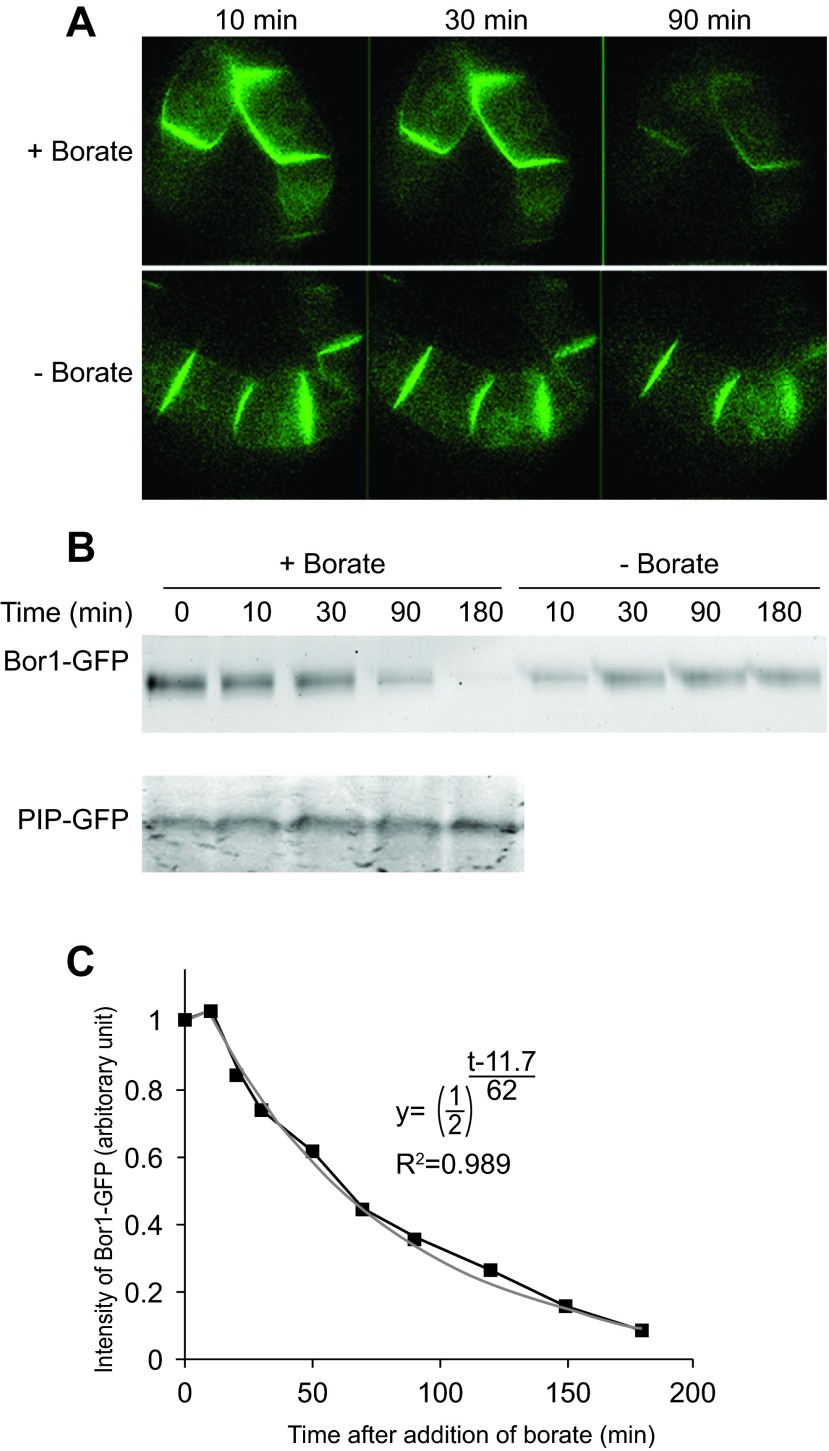
Higher concentrations of borate in the medium induced the degradation of BOR1-GFP. **A**. Fluorescence images of transformed cells expressing BOR1-GFP in medium containing a high concentration of borate (5 mM; +Borate) and a normal concentration of borate (0.1 mM, -Borate). Images were collected with identical exposure conditions using a confocal microscope. **B**. Time course of the decrease in BOR1-GFP signal in the presence of borate. Upper gel; cells expressing BOPR1-GFP were incubated in medium with normal or high borate concentrations for the indicated times. Lower gel; cells expressing PIP-GFP were incubated in high borate medium for the indicated times. Fluorescence of GFP fusion proteins after separation by SDS-PAGE was detected by scanning the gel with an image analyzer. A representative gel image is shown. **C**. Quantification of the degradation of BOR1-GFP. Means of three independent experiments are shown. The gray line is a best-fit curve of the exponential decrease of BOR1-GFP.

We then treated the transformed cells with 5 mM borate for up to 90 min and the fluorescence of BOR1-GFP was monitored under an epifluorescence microscope ( Figure 3A). We found that the BOR1-GFP signal at the peripheral part of the cells decreased significantly during incubation. The same decrease in signal was also observed in the presence of 3 mM borate. Interestingly, lower concentrations of borate (0.1 mM) did not cause a loss of the BOR1-GFP signal. This decrease is specific for BOR1-GFP as the same treatment did not decrease the fluorescence of plasma membrane intrinsic protein (PIP)-GFP, which is a plasma membrane aquaporin fused with GFP.

We also analyzed the fate of BOR1-GFP after separation of cellular proteins by SDS/PAGE and recorded the fluorescence ( Figure 3B). We found that BOR1-GFP signal was decreased in the presence of higher concentrations of borate. This decrease was specific for BOR1-GFP as this protein was not decreased without exposure to borate and the level of PIP-GFP was not changed even with high concentrations of borate. Quantification of the intensities of the BOR1-GFP band allowed us to estimate the kinetic parameters of the decline in the signal intensity. The decrease ratio fitted well into an index recurrence curve with a lag time of 11.7 min and half life of 62 min ( Figure 3C), suggesting that the degradation kinetics is first order. The presence of a lag time suggested that several steps of cellular events must have taken place before degradation of the BOR1-GFP.

### Polarized localization of BOR1-GFP

**Figure 4.**
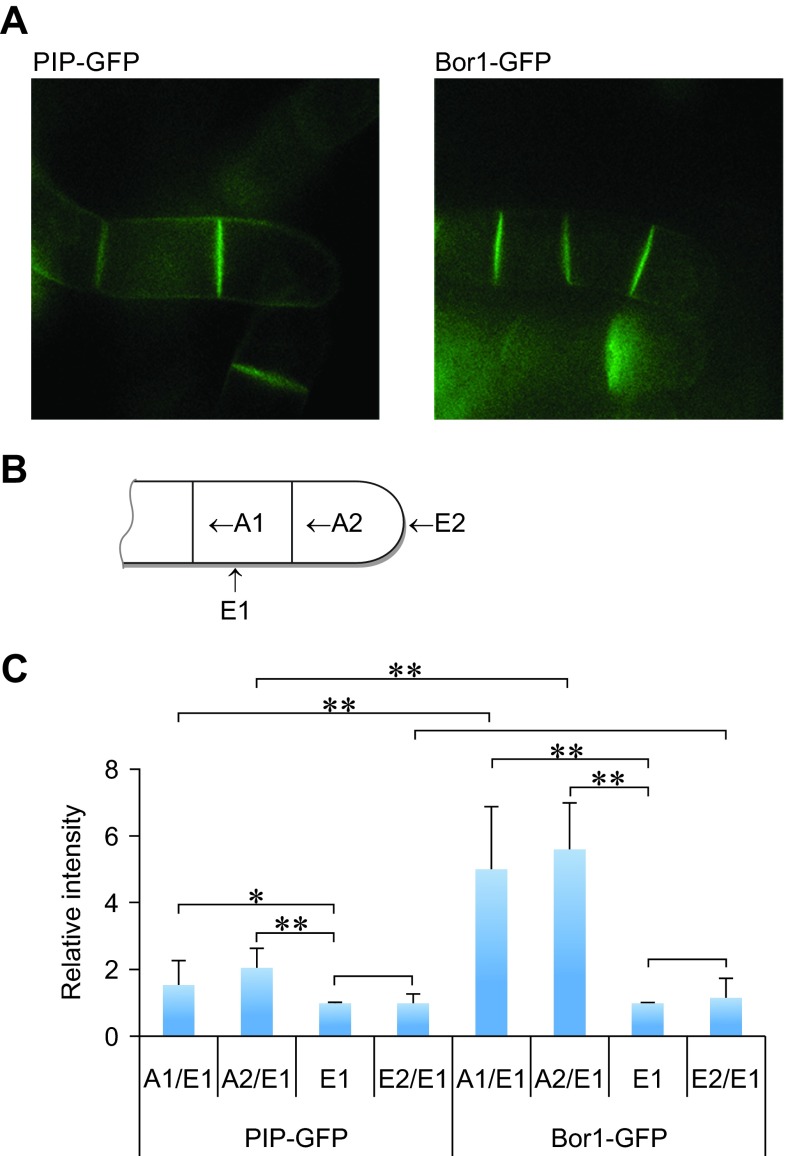
Polarized localization of BOR1-GFP in tobacco BY-2 cells. **A**. Confocal images of PIP-GFP and BOR1-GFP at the four-cell stage. About half to two-thirds of cell clumps are shown. GFP fluourescence of PIP-FGP at the plasma membrane that faces to the medium showed much higher intensities than that of BOR1-GFP. **B**. Schematic representation of each cell membrane face in the cell clump. Only half of the 4-cell stage cell clump was indicated. A1, A2, E1 and E2 indicate the face of cells defined for the analysis of polarized localization. **C**. Comparison of the relative density of fluorescence at each face of PIP-GFP and BOR1-GFP expressing cells. Means (with SD) density relative to the E1 face (n=8) are shown.

As described above, BOR1-GFP but not PIP-GFP was degraded upon addition of borate. In *Arabidopsis* roots BOR1-GFP behavior differs from some other plasma membrane proteins in both borate-dependent degradation and polarized localization in cells ^7, 8^. To address whether localization of BOR1-GFP and PIP-GFP differs in tobacco BY-2 cells, we compared the localization of these proteins in tobacco BY-2 cells at the early log-phase stage ( Figure 4).

With our culture conditions, BY-2 cells at the stationary phase are largely unicellular. Cells at this stage are approximately three-times longer than cells at mid-log phase. When such cells are transferred to fresh medium, they start to divide at the center resulting in two daughter cells which elongate slightly and further divide at the center of each daughter cell without separation. At this stage, a linearly attached clump of cells contain four cells. The clump has four cell walls with a distinct location and age; the one between the two cells that generated at the first cell division, the one between the two cells that generated at the second cell division, the one exposed to the culture medium in the center of two cells, and the one exposed to medium of the two extreme cells containing tips of the cell wall clump. We name these four faces of cells as A1, A2, E1 and E2, respectively ( Figure 4B).

We measured the density of the green fluorescence of these four faces from cells expressing BOR1-GFP and PIP-GFP and the distribution of their relative density (relative to the E1 face). In both cases, the fluorescence density at E1 and E2 was almost the same but in the case of PIP-GFP, the densities at A1 and A2 were approximately 1.5- and 2-fold higher than that of E1, respectively ( Figure 4C). These fluorescence intensities between cells suggest that PIP-GFP is distributed nearly evenly in the plasma membrane as the A1 and A2 faces have two sheets of plasma membrane. In contrast, the density of BOR1-GFP at both A1 and A2 faces were approximately 5-fold higher than that of E1 ( Figure 4C). This observation indicated the polarized localization of BOR1-GFP and clearly showed that this fusion protein accumulates to the plasma membrane that is facing neighboring cells in growing tobacco BY-2 cells.

### Inhibition of protein synthesis partially suppresses borate-induced degradation

The observation that BOR1-GFP is degraded upon addition of borate in the medium ( Figure 3) suggest two possible scenarios for the borate-dependent decrease in BOR1-GFP. The first possible case is that the decrease of BOR1-GFP in the presence of high concentrations of borate is the result of inducible degradation. Another possibility is that the rate of the degradation of BOR1-GFP is rapid in BY-2 cells even in the absence of borate but a high rate of biosynthesis of BOR1-GFP in the normal medium maintained the level of the transporter.

**Figure 5.**
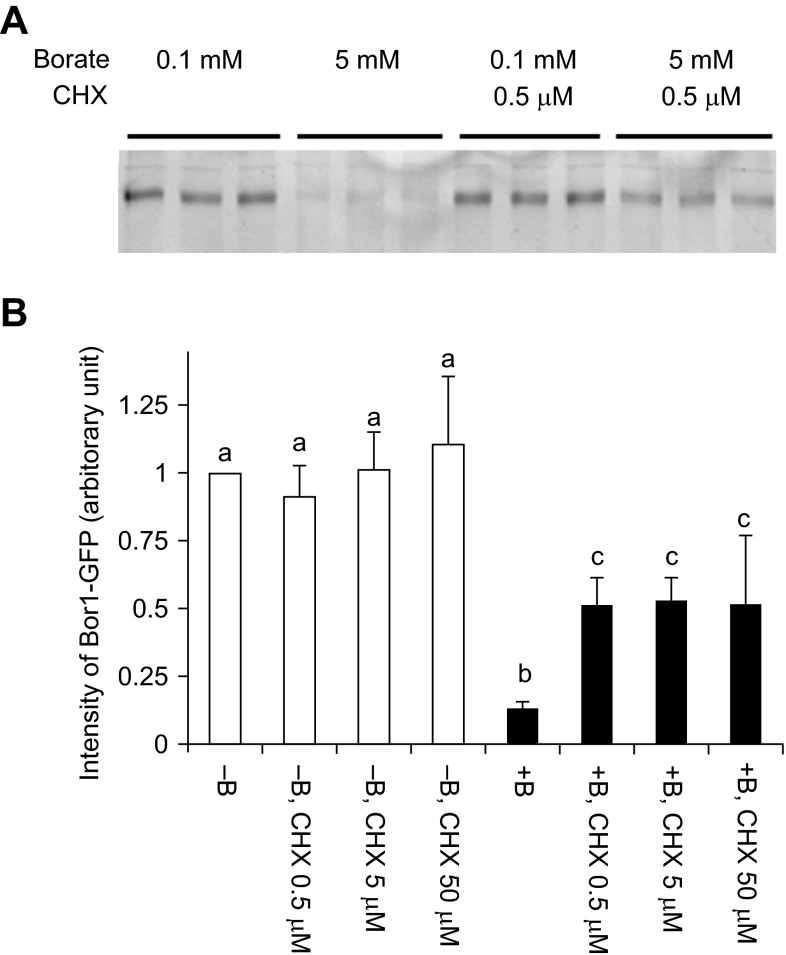
Cycloheximide partially inhibited the borate-dependent degradation of BOR1-GFP. Cells were incubated with normal (0.1 mM) or high (5 mM) concentrations of borate with or without cycloheximide for 2 hours. **A**. Image of BOR1-GFP fluorescence after separation by SDS-PAGE. CHX: cycloheximide. **B**. Quantified result of A. Means of three independent triplicated experiments plus standard deviations are shown a, b and c above the error bars indicate that values in each letter showed significant difference whereas marked with the same letter showed no significant difference (p<0.01) using Tukey’s test.

To address the likely scenario causing the BOR1-GFP decrease in tobacco cells under high borate conditions, we treated the transformed cells with a protein biosynthesis inhibitor cycloheximide (CHX) in either normal or high-boron medium and monitored the level of BOR1-GFP ( Figure 5). In the normal borate medium, addition of CHX did not change the level of BOR1-GFP significantly over 2 hours. This observation indicated that the turnover of BOR1-GFP in normal medium is slow and most of the BOR1-GFP showing the green fluorescence is the result of the accumulation of this protein. This also indicated that the decrease in BOR1-GFP in high borate medium is a result of inducible degradation.

We observed partial inhibition of BOR1-GFP degradation in the presence of CHX ( Figure 5). This observation indicated that continuous synthesis of degradation machinery is necessary for the continuation of the degradation. The partial suppression of the degradation also indicated that the machinery for the degradation is already present in cells that were cultured in the normal medium.

### Endocytosis of BOR1-GFP under high boron condition

**Figure 6.**
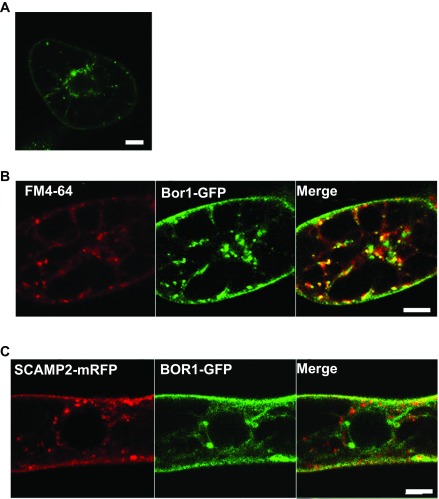
Endocytotic transport of BOR1-GFP during borate-induced degradation. **A**. Two hours after incubating the cells in medium containing 3 mM borate, cells were incubated with FM4-64 for 30 min and analyzed with a confocal fluorescent microscope. **B**. Many, but not all intracellular BOR1-GFP dots colocalized with puncta of FM4-64. **C**. BOR1-GFP did not pass through the trans-Golgi network (TGN) during degradation. BOR1-GFP was transiently expressed in cells expressing SCAMP2-mRFP, which is a marker for TGN and the secretory vesicle cluster ^14^. Co-expressed cells were incubated in medium containing 3 mM borate for 2 hours and the fluorescence was analyzed by confocal laser scanning microscopy. No clear overlap of the intracellular puncta of SCAMP2-mRFP and BOR1-GFP was seen.

It was shown previously that endocytosis is the first step of BOR1-GFP degradation in *Arabidopsis* ^7, 8^. To assess whether similar machinery operates in tobacco BY-2 cells in the presence of high concentrations of borate, we first analyzed the BOR1-GFP signal in cells cultured for 1 to 2 hours under high borate conditions using a confocal laser scanning microscope (CLSM). We found intracellular puncta in cells treated with high borate ( Figure 6A). We then analyzed the colocalization of the BOR1-GFP signal with an early endosome that can be marked with FM4-64. Tobacco cells expressing BOR1-GFP were incubated for 2 hours in a medium containing 3 mM borate and then the cells were stained with FM4-64 on ice, washed with cold medium containing 3 mM borate, after which they were returned to room temperature to resume intracellular trafficking as described ^14^. Five to 10 min after FM4-64 incubation, dotted structures emitting red fluorescence, which represent an early endosome distinct from the *trans*-Golgi network (TGN) in tobacco cells ^14^, was observed ( Figure 6B, left). Most of the early endosome signal colocalized with intracellular dots of BOR1-GFP, suggesting that BOR1-GFP passes through early endosome during degradation as in the case of *Arabidopsis* cells.

It is also known that FM4-64 is transported from the early endosome to the TGN, and thereafter targeted to the vacuolar membrane. To evaluate whether BOR1-GFP passes through the TGN, we expressed SCAMP2-mRFP ^14^ in cells expressing BOR1-GFP. SCAMP2-mRFP localizes in the plasma membrane, the TGN and the secretory vesicle cluster, which is an exocytotic compartment derived from the TGN ^14^. No colocalization of green and red intracellular dots was observed when cells were treated with 3 mM borate for 2 hours ( Figure 6C). These findings suggested that the BOR1-GFP is internalized to endosomes and then sorted to a degradation pathway from the endocytotic route, which is directed to the TGN.

### Traffic and other inhibitors that affect the degradation

It was previously reported that the treatment of *Arabidopsis* roots with brefeldin A (BFA), which is an inhibitor for both endocytosis and exocytosis, prevents the borate-dependent degradation of BOR1-GFP ^7^. To address whether this compound is also effective in tobacco BY-2 cells and to assess the mechanism of the degradation, we analyzed borate-induced degradation in the presence of various inhibitors ( Figure 6). We used BFA, 2,3,5-triiodobenzoic acid (TIBA), a compound that stabilizes the actin bundle ^15^; tyrphostin A23, which is a tyrosine kinase inhibitor, which can inhibit endocytosis by competing with the interaction of the AP-2 adaptor complex and Tyr-containing endocytosis motifs in mammalian cells and also can inhibit the endocytosis of rice SCAMP1-YFP and FM4-64 in tobacco BY-2 cells ^16, 17^; ikarugamycin, which is an inhibitor of clathrin-mediated endocytosis in tobacco ^18^; colchicine, which is an inhibitor of microtubule depolymerization; latrunculin B, which inhibits actin polymerization; 2,3-butanedione monoxime (BDM), which is a general myosin ATPase inhibitor and inhibits the endocytosis of SCAMP2-mRFP in tobacco BY-2 cells ^14^; MG-132, which is an inhibitor of proteasomes at low concentrations and also inhibits other proteases; and K-252a, which is a general Ser/Thr type protein kinase inhibitor.

**Figure 7.**
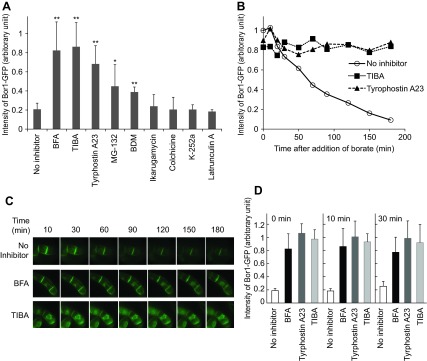
Inhibitors that prevent borate-dependent degradation. **A**. Cells incubated with or without inhibitors for 30 min were further incubated in medium containing 5 mM of borate for two hours in the presence of the same inhibitors. The relative intensity of BOR1-GFP fluorescence was quantified as described in the Figure 3 legend. The final concentration of inhibitors in the culture and the number of replicates were; brefeldin A (BFA), 25 μM, n=8; 2,3,5-triiodobenzoic acid (TIBA), 50 μM, n=5; tyrphostin n=5; Tyrphostin A23, 100 μM, n=5; MG-132, 400 μM, n=5; 2,3-butanedione monoxime (BDM), 40 mM, n=3; K-252a, 4 μM, n=3; Ikarugamycin, 20 μM, n=3; Latrunculin A, 100 μM, n=3; colchicin, 200 μM, n=3. Error bars, SD. ** and * represent the significant difference relative to no inhibitor (p<0.01 and <0.05 respectively). **B**. Time-course analysis of the degradation of BOR1-GFP in the presence of either TIBA, tyrphostin A23 or in the absence of an inhibitor. **C**. Changes in the fluorescence pattern of BOR1-GFP in the absence and presence of BFA or TIBA in the presence of 5 mM borate. **D**. Timing of the addition of inhibitors did not affect the subsequent degradation. Cells were incubated in medium containing 5 mM of borate. At the same time (0 min) or the other indicated times, either BFA, tyrphostin A23 or TIBA was included in the medium and further incubated for up to 120 min. The intensity of the BOR1-GFP band relative to that at the time when the inhibitor was included is shown. Error bars, SD.

Three hours after application of inhibitor solution to the tobacco cell culture, borate was added at a final concentration of 5 mM and further incubated for 2 hours. Thereafter, the amount of BOR1-GFP was quantified ( Figure 7A). We found that BFA and TIBA inhibited degradation almost completely (>80% remained) at a concentration that inhibits the endocytosis of SCAMP2-YFP ^14^. A slightly weaker effect of tyrphostin A23 was observed at 400 μM, which is a concentration similar to that used in the works of Leborgne-Castel *et al.* ^16^ and Lam *et al.* ^17^. In this case, approximately 70% of BOR1-GFP remained in the cell. We also found partial inhibition of degradation by BDM at 5 mM, the concentration that inhibits the endocytosis of SCAMP2-YFP ^14^. We also observed partial inhibition by MG-132 at 400 μM. In these two cases, approximately 40% of BOR1-GFP remained in the cell. Other inhibitors tested did not cause any detectable effect on the extent of degradation.

Time-course analysis of the decrease of BOR1-GFP in the presence of TIBA and tyrphostin A23 ( Figure 7B) clearly showed that most of the degradation was inhibited up to 3 hours. We next addressed whether the inhibition occurred at the point of endocytosis from the plasma membrane or at intermediate compartments. Fluorescence images were collected in transformed tobacco cells in the presence of both inhibitors and borate. We found that the images were not significantly different up to 3 hours in the presence of either BFA or TIBA ( Figure 7C). These observations suggested that both BFA and TIBA inhibited the early event of endocytosis. We could not analyze fluorescence images in the presence of tyrphostin A23 because treatment of BY-2 cells with this compound increased cellular fluorescence significantly and such fluorescence was independent of the expression of BOR1-GFP.

To address whether the inhibition of endocytosis required BY-2 cells to be pretreated with BFA, tyrphostin A23 or TIBA, we investigated the timing of adding the inhibitors ( Figure 7D). We compared the level of BOR1-GFP without inhibitors with BOR1-GFP levels when these inhibitors were added into the culture. We found that addition of inhibitors at the same time, in the lag time as well as during the degradation phase, prevented the decrease in BOR1-GFP almost completely. These observations indicated that these inhibitors have a direct effect against the endocytosis of BOR1-GFP and were not a side effect of these chemicals. These observations also suggested that actin dynamics, GDP-GTP exchange of Arf small GTPase, and either tyrosine phosphorylation or AP-2 adaptor interaction are necessary for the degradation of BOP1-GFP in tobacco BY-2 cells.

### Tyrosine mutations prevented borate-induced degradation

**Figure 8.**
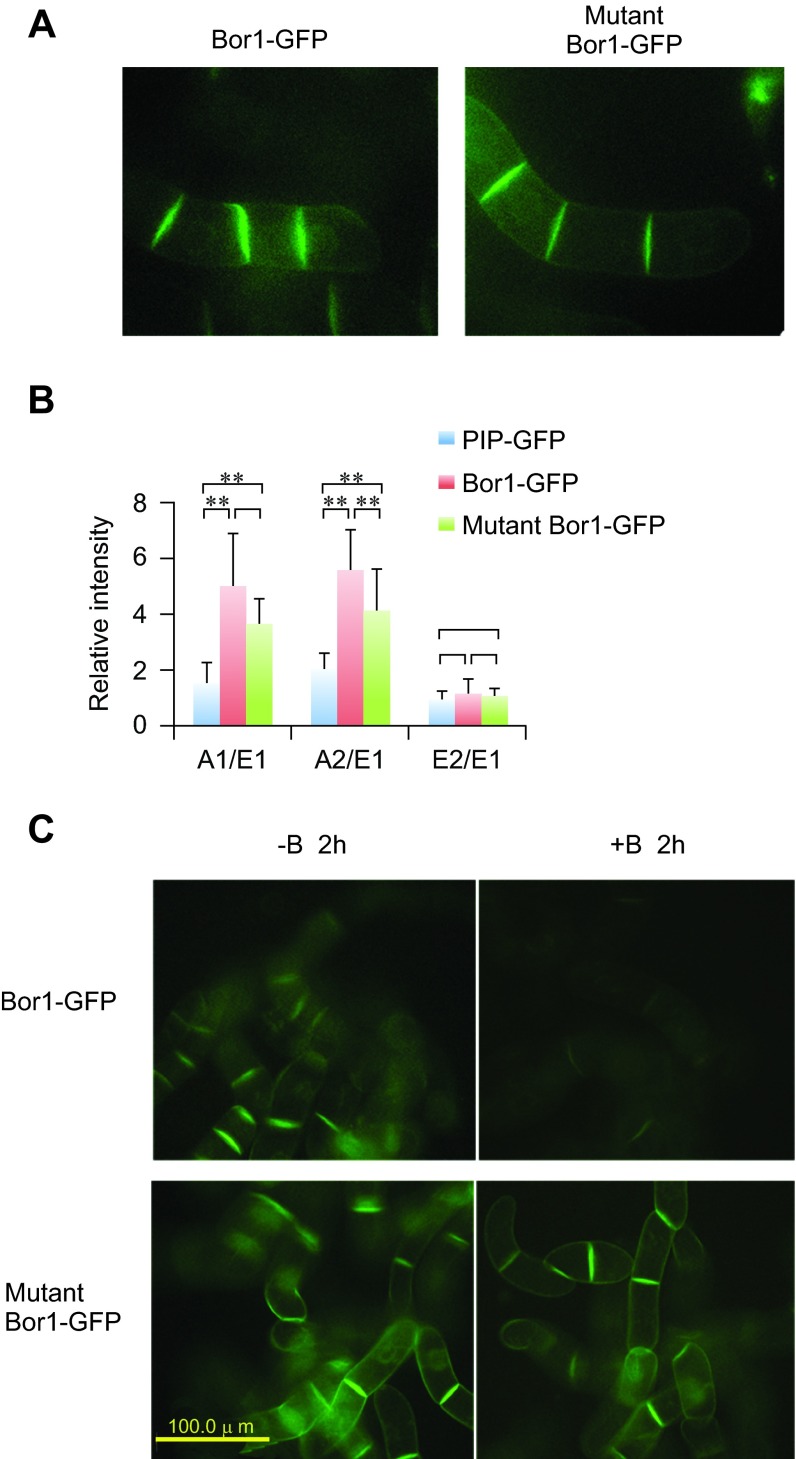
Expression of Tyr mutant of BOR1-GFP. **A**. Confocal images of 4-cell stage tobacco cells expressing wild-type and mutant BOR1-GFP. **B**. Comparison of the relative density of fluorescence at each face of the cells expressing either PIP-GFP, wild-type BOR1-GFP or mutant BOR1-GFP. Mean densities relative to the E1 face (n=8) are shown. Error bars, SD. ** represents significance at p<0.01. **C**. Mutant BOR1-GFP is stable in the presence of high-concentrations of borate. Epifluorescence images of cells incubated with normal (-B, 0.1 mM) or high borate (+B, 5 mM) for 2 h are shown.

It was shown that both polarized localization and borate-dependent degradation are mediated by specific Tyr residues in BOR1-GFP in *Arabidopsis* root cells ^8^. To address whether these Tyr residues also contribute to the localization and borate-induced degradation of BOR1-GFP in tobacco BY-2 cells, we expressed Y373A/Y398A/Y405A mutants of BOR1-GFP in BY-2 cells ( Figure 8). Confocal images of the mutant BOR1-GFP displayed higher intensity of fluorescence signals between attached cells as in the case of wild-type BOR1-GFP ( Figure 8A). Quantification of the intensities of different faces indicated that this protein also showed a polar localization, although the polarized nature seems somewhat weaker than the wild-type ( Figure 8B). This observation indicated that the polarized localization of BOR1-GFP in tobacco cells is largely independent of the tyrosine residues in the BOR1 protein.

When tobacco cells expressing the mutant protein were incubated in medium containing 5 mM borate, we still observed strong fluorescence of GFP signals. We did not detect any difference in the pattern and intensity of the fluorescence signals between cells incubated in normal medium and high borate medium ( Figure 8C). Under the same condition, most of the wild-type BOR1-GFP was degraded in the cell ( Figure 3, Figure 8C). This observation indicated that Y373, Y398, and/or Y405 residues of BOR1-GFP are involved in borate-induced degradation in tobacco BY-2 cells as in the case in *Arabidopsis* root cells.

Localization and degradation data of the Arabidopsis borate transporter BOR1 in tobacco BY-2 cells Borate effect on BY-2 growth.csv
This is the dataset for Figure 2 in the associated article. Seven-day old tobacco BY-2 cells were subcultured into medium containing the indicated concentration of borate (mM) and shaken for a week. The volume of cells in the culture was measured after sedimentation of the cells by centrifugation. Packed cell volume indicate the actual volume of sedimented cells (ml) and the relative value represent the relative volume of the average of the triplicated experiment at 0.1 mM borate condition, which corresponds to the normal growth condition. Average of relative value and STDEV were used to make Figure 2.
BOR1-GFP localization in BY-2 cells.csv
This is the dataset for Figures 4C and 8B in the associated research article. The column A “cell type” indicates the cell line and collected image name. PG represents the PIP-GFP, WT represent the wild-type BOR-GFP, and mut represent the mutant BOR-GFP. The numbers after PG, WT and mut indicate the image number. Column B to I represent the actual data collected for the analysis. At each face of the cell images a box was made on the region corresponds to either of the face of the cells and the density of the intensity in the box was quantified (e.g. column B, A1). The same size box was copied close to the box at the outside of the cell and used to quantify the background intensity and the density of the intensity in the box was quantified (e. g. column C, A1 BG). Column J to M represent the subtracted density of fluorescence from the face to background (e. g. column B value minus column C value). The coumn N to Q represent the relative value of intensity to E1 face. The A1, A2, E1, E2 faces are indicated in Figure 4B.
Borate medium conc BOR1-GFP degradation.csv
This is the dataset for Figure 3C in the associated research article. Time course analysis of the degradation of BOR-GFP was quantified from the gel image. The column A indicate the experiment No. and the relative value of each experiments to time 0 as well as the average and standard deviation (STDEV) value of the relative intensity to time 0. From column B to K the top row indicate the time point and the other row indicate the actual and relative values, average and STDEV of relative to 0 time intensity.
CHX BOR1-GFP degradation.csv
This is the dataset for Figure 5B. Images were quantified from the gel and the relative value to without borate was calculated as described in the legend to <Borate medium conc BOR1-GFP degradation.csv>. Column A is the experiment No.
Colum B to I are the relative values of each treatment described in row A of the column. The numbers in the top row of columns B to I are the concentration of cyclohexamide (mM).
Time course inhibitors BOR1-GFP degradation.csv
This is the dataset for Figure 7B in the associated research article. In each experiment, to analyze the effect of TIBA or tyrphostin A23, a control without both borate and inhibitor was used. The relative value of the intensity of the bands were quantified as in the legend to <Borate medium conc BOR1-GFP degradation.csv>. The values of row 1-4 are identical to the relative value of <Borate medium conc BOR1-GFP degradation.csv>.
Borate dependent degradation inhibition.csv
This is the dataset for Figure 7A in the associated article. Column A indicates the experiment No. and the average and STDEV of the relative value. Column B indicates whether the data is the actual intensity or is the relative intensity to the values without both borate and inhibitors. The top row of column C to O indicates the presence and absence of borate and the names of inhibitors used. From the second to 25th row of columns C to O, the actual value as well as the relative values for without both borate and inhibitors were shown.
Relative amount of Bor1-GFP at the addition of inhibitor (from time-course data).csv
This is the background dataset for the processing of the data for Figure 7D in the associated research article. At the zero time or indicated time inhibitors were add to the culture. The relative intensity of bands to the start of the incubation with borate (0 time) is shown. Based on the average value in this dataset the relative value of the intensities of the bands in <Timimg of the addition of inhibitors (Fig 7D).csv> were calculated.
Timimg of the addition of inhibitors (Fig 7D).csv
This is the dataset for Figure 7D in the associated article. The relative values against each time points are shown. The time written in the first row indicates the timing of the addition of inhibitors. After calculation of the relative intensity to the time 0 without borate value as calculated in <BOR1-GFP localization in BY-2 cells.csv>, the average and STDEV of three experiments were calculated. Thereafter these values were normalized to the average value calculated in <Relative amount of Bor1-GFP at the addition of inhibitor (from time-course data).csv> (rows 7 and 8) and used to make Figure 7D.

## Discussion

We found that the *Arabidopsis* borate transporter BOR1-GFP fusion protein was targeted to the plasma membrane of rapidly growing tobacco BY-2 cells. The plasma membrane localization of BOR1-GFP was different from that of PIP-GFP. BOR1-GFP was more abundant in the plasma membranes that face neighboring cells ( Figure 4A, B). This observation indicates that BOR1-GFP showed a polarized localization in tobacco BY-2 cells. This observation further indicates that plasma membranes of BY-2 cells facing the medium and the neighboring cells are distinct in nature. It was shown in *Arabidopsis* root cells that BOR1-GFP is localized in the proximal region of the plasma membrane. However, polarized localization in BY-2 cells and *Arabidopsis* root cells seems to be mediated by distinct mechanisms because the latter, but not the former, is sensitive to the mutation of Tyr residues in the cytoplasmic loop of BOR1 protein ( Figure 8A, B) ^8^.

The concentrations of borate that caused the degradation of BOR1-GFP in BY-2 cells was >3 mM, which is higher than that in *Arabidopsis* plants (100 μM) ^7^. This difference might correlate with the difference of high-boron sensitivity of the growth between the two species. BY-2 cells can grow normally up to 5 mM borate ( Figure 2D) whereas 300 μM borate caused about 50% reduction of the growth of *Arabidopsis* ^19^. Thus it will be interesting to investigate in the future what attributes of plants including tobacco cells and *Arabidopsis* plants determine sensitivity to high borate levels.

The borate-induced degradation of BOR1-GFP was inhibited by the protein synthesis inhibitor CHX ( Figure 5). We observed previously that CHX treatment did not inhibit endocytosis of SCAMP2-YFP in tobacco BY-2 cells ^14^. Therefore, the effect of CHX on the inhibition of BOR1-GFP is not a general response in tobacco cells. The inhibition by CHX on BOR1-GFP degradation was not complete and approximately 50% of the protein was degraded under CHX treatment ( Figure 5). This observation suggested that BY-2 cells used pre-existing machinery for the degradation of BOR1-GFP. However, the degradation the machinery was limited in the cell and *de novo* synthesis of the machinery is required for the continuation of the degradation.

The plasma membrane accumulation of BOR1-GFP was abolished when cells were incubated in medium containing high concentrations of borate. The decline in the BOR1-GFP was mediated by endocytosis and further degradation ( Figure 3 and Figure 6) and was dependent on the tyrosine residues ( Figure 8C) as observed in *Arabidopsis* root cells. These observations suggest that boron sensing mechanisms as well as the borate-dependent induction of the endocytosis of BOR1-GFP is conserved in different plant species. However, there are some differences in the endocytotic trafficking between tobacco cells and *Arabidopsis* root cells. The most pronounced difference is the organization of organelles in these cells. In tobacco BY-2 cells, early endosomes and the TGN are separated ^14^ and BOR1-GFP passed through the early endosome but not the TGN during degradation ( Figure 6B, C). In contrast, early endosomes cannot be distinguished from the TGN in *Arabidopsis* root cells ^9^. Therefore, differentiation of secretory organelles is not completely identical in these two species. Further analysis of the degradation of BOR1-GFP in either cultured *Arabidopsis* cells or tobacco root cells will give a clearer picture as to whether the difference in trafficking paths originate from differences between species or the differentiation state of the cells.

The endocytosis-dependent degradation of BOR1-GFP was inhibited almost completely upon addition of the inhibitors BFA, TIBA and tyrphostin A23. BFA is known as an inhibitor of the GDP-GTP exchange factor for ADP-ribosylation factor and has many effects on different plant cells ^19^. On tobacco BY-2 cells, we have shown that BFA inhibits both endocytosis of SCAMP2-YFP, Golgi protein localization and export from the endoplasmic reticulum ^14, 21, 22^. It was also shown that BFA inhibits the endocytosis of BOR1-GFP in *Arabidopsis* root cells ^7^. The fact that BFA inhibited the endocytosis of BOR1-GFP in tobacco BY-2 cells in the presence of borate ( Figure 7) indicates that some machinery for the borate-dependent endocytotic degradation is conserved between *Arabidopsis* root cells and tobacco BY-2 cells.

TIBA is known to inhibit the endocytosis of the auxin transporter and its function is to stabilize the actin cytoskeleton at 25 μM in tobacco BY-2 cells ^15^. The fact that TIBA at a similar concentration range inhibited the degradation of BOR1-GFP almost completely ( Figure 7) suggested that the actin cytoskeleton is involved in the endocytotic degradation of BOR1-GFP. The partial inhibition of degradation by BDM ( Figure 7), which is an inhibitor of myosin motors ^7^ also supports the idea that an actin-dependent mechanism is a key part of the machinery for the endocytotic degradation of BOR1-GFP.

Another inhibitor that prevented borate-dependent degradation was tyrphostin A23. This chemical is known to inhibit Tyr-signal mediated endocytosis by inhibiting the recognition of the AP-2 adaptor complex signal in mammalian cells. The requirement of Tyr residues in BOR1 for endocytosis ( Figure 8C) agrees well with the action of tyrphostin A23 in mammalian cells. This compound is also known to inhibit endocytosis in several plant species ^16, 17, 23, 24^. It was reported recently that this compound inhibited elicitor-induced endocytosis in tobacco BY-2 cells and this event seems related to the induction of clathrin-mediated endocytosis ^16^. However, ikarugamycin, which is known to inhibit clathrin-mediated endocytosis in both animal cells ^25^ and tobacco protoplasts ^18^, did not inhibit the degradation of BOR1-GFP. It was reported recently that AP-2 is involved not only in clathrin-mediated endocytosis but also in clathrin-independent endocytosis in mammalian cells ^26^. Therefore further investigation is necessary to elucidate whether clathrin is involved in the endocytosis of BOR1-GFP.

In addition to these inhibitors, we observed that MG-132 also inhibited the degradation of BOR1-GFP. It was reported that this compound also inhibits the degradation of interleukin-2 through the inhibition of endocytosis of its receptor in mammalian cells ^27^. As MG-132 is a potent inhibitor of proteasomes, our finding raises the possibility that a proteasome system is somehow involved in BOR1-GFP degradation. However, our data does not rule out the possibility that the target of MG-132 on BOR1-GFP degradation is different from proteasomes as we needed a very high concentration (400 μM) of this compound to prevent BOR1 degradation and MG-132 can inhibit other proteases, such as calpain in animals, at a high concentration ^28^. Thus, future experiments will be necessary to ascertain whether proteasomes are involved in the degradation of BOR1-GFP and to show which molecule is the real target of MG-132 on BOR1-GFP degradation in tobacco cells.

## Materials and methods

### Plasmids

Plasmids for the expression of wild-type and mutant BOR1-GFP, and SCAMP2-mRFP have been described previously ^7, 8, 14^. The expression plasmid for PIP-GFP was a kind gift from Dr. M. Maeshima (Nagoya University). This plasmid contains radish plasma membrane type aquaporin PAQ1 ^29^ and GFP fusion protein under an enhancer-duplicated cauliflower mosaic virus 35S promoter ^30^.

### Chemicals

The following chemicals were used to test the inhibition of the degradation: BFA (Wako Pure Chemicals, Osaka, Japan), CHX (Wako Pure Chemicals, Osaka, Japan), TIBA (Aldrich Chem. Co. USA), Tyrphostin A23 (Sigma-Aldrich, Inc, Missouri, USA), colchicine (Tokyo Kasei Co., Tokyo, Japan), K-252a (Sigma-Aldrich, Inc, Missouri, USA), BDM (Sigma-Aldrich, Inc., Missouri, USA), Latruncurin A (Calbiochem, San Diego, USA), ikarugamycin (BIOMOL Research Labs Inc. USA), MG-132 (Calbiochem Inc. San Diego USA), wortmannin (Calbiochem Inc. San Diego USA), 3MA (Sigma-Aldrich, Inc., Missouri, USA) and E-64 (Peptide Institute, Osaka, JAPAN).

### Cell culture and transformation

The culture of tobacco BY-2 cells (a kind gift from Drs. K. Nakamura (Nagoya University) and T. Nagata (University of Tokyo) and the transformation of this cell line were as described ^30^. Cells were cultured weekly and 3 or 4 day-old cultures were used unless otherwise stated. For transient transformation of cells expressing BOR1-GFP to express SCAMP2-mRFP in the same cell, *Agrobacterium tumefaciens EHA105* ^31^ harboring a binary plasmid was co-cultured with BOR1-GFP expressing tobacco cells for 2 days, washed with fresh medium and further incubated in medium containing 200 mg/L cefotaxime for a day. Thereafter fluorescence of cells was visualized using CLSM.

### Quantification of GFP fusion proteins

Cells expressing GFP fusion proteins were lysed with equal volumes of buffer consisting of 0.4 M Sorbitol, 0.1 M KOAc, 6 mM EGTA, 4 mM EDTA, 40 mM HEPES-KOH, 2 mM DTT, 1 drop/100 ml protease inhibitor tablet (Complete EDTA-free, Roche), pH 7.3 at room temperature. Cells were lysed by sonication and the resulted cell lysate was centrifuged at 300 × g for 10 min. The resulting supernatant was used as the cell lysate. Proteins in the cell lysate was mixed with 1/5 volume of a buffer consisting 0.25 M Tris-HCl, 0.5 M dithiothreitol, 50 % glycerol, 10 % SDS, 0.025 % bromophenol blue, pH 6.8 and loaded on SDS-polyacrylamide gels without heating. Fluorescence images of the gels were recorded using Typhoon 9600 scanner (GE Healthcare, Tokyo, Japan) at the following setting; filter 520SPCy2, excitation 488 nm blue laser at PMT 650, medium sensitivity). The intensities of bands were quantified using Image Quant software (GE Healthcare).

### Microscopic analysis

For epifluorescence detection, cells were mounted on glass slides and observed using IX40 inverted microscope with an LCPlanFL 20X lens using NBY excitation/emission filter set and a DP-70 camera (Olympus Co. Tokyo Japan). For time-lapse image collection, cell suspension was placed into in a well of glass-bottom 96 well micro-plates (EZVIEW® Glass Bottom Culture Plates LB, Asahi Techno Glass Co. Tokyo, Japan). Cells in the plate wells were observed using an IX80 inverted fluorescence microscope equipped with DSU spinning disk confocal unit and an Uplan SApo 40×/0.90 lens using NBY or RFP excitation/emission filter sets (Olympus). Images were collected using an iXON+-DU888E-CS0-BV camera (Andor) under the control of Meta Imaging Series 7.6.3 software (Molecular Devices Inc. Sunnyvale, CA, USA).

For the colocalization analysis, cells were mounted on slides with culture medium. These cells were observed using a CLSM system (LSM510 META, Axioplan2 Imaging; Carl Zeiss) with a Plan-Apochromat lens (633 1.4 oil differential interference contrast; optical slices of 1 mm). We used a 25-mW argon laser (power, 5%) with 488-nm excitation and a 505–530-nm band-pass filter for GFP8. For FM4-64 (Molecular Probes, Eugen, USA) we used a 650-nm long-pass filter and 488-nm excitation. A 560-nm long-pass filter and 1-mW He-Ne laser at 80% power with 543-nm excitation was used for Alexa Fluor 568 and mRFP. Crosstalk was prevented using a multitrack configuration with line sequential scanning. Composite figures were prepared using Zeiss LSM Image Browser software.

For the measurement of intensity, confocal images at 256 bit tiff file was quantified using ImageJ software. If required, relative density to the E1 face was calculated in each cell clump.

### Borate treatment to monitor endocytosis

An aliquot of 0.5 M boric acid in water was added to 96.5 ml of suspension culture of three-day old tobacco cells expressing BOR1-GFP in a 300 ml Erlenmeyer flask at a concentration of 3 or 5 mM (as indicated in the legends to figures) and further cultured with the same condition of regular culture. For the control, cells grown in a normal culture medium, which contains 0.1 mM borate, was used. At the indicated time, a 0.5 or 1 ml aliquot of the culture was removed from the flask and used for the analysis.

### Statistical analyses

For the comparison of the two values student’ t-test was employed using Microsoft Excel software. For the comparison of difference with multi values, Tukey’s test was used.

## References

[1] Marschner H: Mineral nutrition of higher plants, 2nd Ed. Academic Press, San Diego.1995 Reference Source

[2] Nable R Banuelos G Paull J: Boron toxicity. *Plant Soil.* 1997;193:181–191 Reference Source

[3] Takano J Miwa K Fujiwara T: Boron transport mechanisms: Collaboration of channels and transporters. *Trends Plant Sci.* 2008;13(8):451–457 10.1016/j.tplants.2008.05.007 18603465

[4] Takano J Noguchi K Yasumori M: *Arabidopsis* boron transporter for xylem loading. *Nature.* 2002;420(6913):337–340 10.1038/nature01139 12447444

[5] Miwa K Takano J Omori H: Plants tolerant of high boron levels. *Science.* 2007;318(5855):1417 10.1126/science.1146634 18048682

[6] Sutton T Baumann U Hayes J: Boron-toxicity tolerance in barley arising from efflux transporter amplification. *Science.* 2007;318(5855):1446–1449 10.1126/science.1146853 18048688

[7] Takano J Miwa K Yuan L: Endocytosis and degradation of BOR1, a boron transporter of *Arabidopsis thaliana*, regulated by boron availability. *Proc Natl Acad Sci U S A.* 2005;102(34):12276–12281 10.1073/pnas.0502060102 16103374 PMC1189310

[8] Takano J Tanaka M Toyoda A: Polar localization and degradation of Arabidopsis boron transporters through distinct trafficking pathways. *Proc Natl Acad Sci U S A.* 2010;107(11):5220–5225 10.1073/pnas.0910744107 20194745 PMC2841934

[9] Yoshinari A Kasai K Fujiwara T: Polar localization and endocytic degradation of a boron transporter, BOR1, is dependent on specific tyrosine residues. *Plant Signal Behav.* 2012;7(1):46–49 10.4161/psb.7.1.18527 22301967 PMC3357367

[10] Viotti C Bubeck J Stierhof YD: Endocytic and secretory traffic in Arabidopsis merge in the trans-Golgi network/early endosome, an independent and highly dynamic organelle. *Plant Cell.* 2010;22(4):1344–1357 10.1105/tpc.109.072637 20435907 PMC2879741

[11] Kasai K Takano J Miwa K: High boron-induced ubiquitination regulates vacuolar sorting of the BOR1 borate transporter in *Arabidopsis thaliana*. *J Biol Chem.* 2011;286(8):6175–6183 10.1074/jbc.M110.184929 21148314 PMC3057829

[12] Nakagawa Y Hanaoka H Kobayashi M: Cell-type specificity of the expression of Os BOR1, a rice efflux boron transporter gene, is regulated in response to boron availability for efficient boron uptake and xylem loading. *Plant Cell.* 2007;19(8):2624–2635 10.1105/tpc.106.049015 17675406 PMC2002629

[13] Matsuoka K: Protein sorting and protein modification along the secretory pathway in BY-2 cells. *In.*T Nagata., S Hasezawa., D Inze., eds Tobacco BY-2 cells: Biotechnology in Agriculture and Forestry, Springer-Verlag, Heidelberg,2004;53:283–298 10.1007/978-3-662-10572-6_19

[14] Toyooka K Goto Y Asatsuma S: A mobile secretory vesicle cluster involved in mass transport from the Golgi to plant cell exterior. *Plant Cell.* 2009;21(4):1212–1229 10.1105/tpc.108.058933 19376937 PMC2685622

[15] Dhonukshe P Grigoriev I Fischer R: Auxin transport inhibitors impair vesicle motility and actin cytoskeleton dynamics in diverse eukaryotes. *Proc Natl Acad Sci U S A.* 2008;105(11):4489–4494 10.1073/pnas.0711414105 18337510 PMC2393819

[16] Leborgne-Castel N Lherminier J Der C: The plant defense elicitor cryptogein stimulates clathrin-mediated endocytosis correlated with reactive oxygen species production in Bright Yellow-2 tobacco cells. *Plant Physiol.* 2008;146(3):1255–1266 10.1104/pp.107.111716 18184734 PMC2259092

[17] Lam SK Cai Y Tse YC: BFA-induced compartments from the Golgi apparatus and trans-Golgi network/early endosome are distinct in plant cells. *Plant J.* 2009;60(5):865–881 10.1111/j.1365-313X.2009.04007.x 19709389

[18] Onelli E Prescianotto-Baschong C Caccianiga M: Clathrin-dependent and independent endocytic pathways in tobacco protoplasts revealed by labelling with charged nanogold. *J Exp Bot.* 2008;59(11):3051–3068 10.1093/jxb/ern154 18603619 PMC2504345

[19] Noguchi K Yasumori M Imai T: *bor1-1*, an *Arabidopsis thaliana* mutant that requires a high level of boron. *Plant Physiol.* 1997;115(3):901–906 10.1104/pp.115.3.901 9390427 PMC158553

[20] Robinson DG Langhans M Saint-Jore-Dupas C: BFA effects are tissue and not just plant specific. *Trends Plant Sci.* 2008;13(8):405–408 10.1016/j.tplants.2008.05.010 18640067

[21] Matsuoka K Watanabe N Nakamura K: O-glycosylation of a precursor to a sweet potato vacuolar protein, sporamin, expressed in tobacco cells. *Plant J.* 1995;8(6):877–889 10.1046/j.1365-313X.1995.8060877.x 8580960

[22] Yuasa K Toyooka K Fukuda H: Membrane-anchored prolyl hydroxylase with an export signal from the endoplasmic reticulum. *Plant J.* 2005;41(1):81–94 10.1111/j.1365-313X.2004.02279.x 15610351

[23] Dhonukshe P Aniento F Hwang I: Clathrin-mediated constitutive endocytosis of PIN auxin efflux carriers in Arabidopsis. *Curr Biol.* 2007;17(6):520–527 10.1016/j.cub.2007.01.052 17306539

[24] Konopkaa CA Backuesb SK Bednarek SY: Dynamics of Arabidopsis dynamin-related protein 1C and a clathrin light chain at the plasma membrane. *Plant Cell.* 2008;20(5):1363–1380 10.1105/tpc.108.059428 18502847 PMC2438457

[25] Luo T Fredericksen B Hasumi K: Human immunodeficiency virus type 1 Nef-induced CD4 cell surface downregulation is inhibited by ikarugamycin. *J Virol.* 2001;75(5):2488–2492 10.1128/JVI.75.5.2488-2492.2001 11160755 PMC114835

[26] Lau AW Chou MM: The adaptor complex AP-2 regulates post-endocytic trafficking through the non-clathrin Arf6–dependent endocytic pathway. *J Cell Sci.* 2008;121(Pt 24):4008–4017 10.1242/jcs.033522 19033387

[27] Yu A Malek TR: The proteasome regulates receptor-mediated endocytosis of interleukin-2. *J Biol Chem.* 2001;276(1):381–385 10.1074/jbc.M007991200 11032838

[28] Mailhes JB Hilliard C Lowery M: MG-132, an inhibitor of proteasomes and calpains, induced inhibition of oocyte maturation and aneuploidy in mouse oocytes. *Cell Chromosome.* 2002;1(1):2 10.1186/1475-9268-1-2 12437781 PMC149371

[29] Suga S Imagawa S Maeshima M: Specificity of the accumulation of mRNAs and proteins of the plasma membrane and tonoplast aquaporins in radish organs. *Planta.* 2001;212(2):294–304 10.1007/s004250000396 11216851

[30] Matsuoka K Nakamura K: Propeptide of a precursor to a plant vacuolar protein required for vacuolar targeting. *Proc Natl Acad Sci U S A.* 1991;88(3):834–838 10.1073/pnas.88.3.834 1992474 PMC50908

[31] Hood EE Kusnadi A Nikolov Z: Molecular farming of industrial proteins from transgenic maize. *Adv Exp Med Biol.* 1999;464:127–147 10335391 10.1007/978-1-4615-4729-7_11

