# Supplementary material for: Polarized localization and borate-dependent degradation of the Arabidopsis borate transporter BOR1 in tobacco BY-2 cells
Source: F1000Res. 2013 Sep 13;2:185. [Version 1] doi: 10.12688/f1000research.2-185.v1 (PMC3954168; doi:10.12688/f1000research.2-185.v1)
Supplement: Peer review history

# Peer review: Referee response for version 1

**Published:** 2013-11-06

**Authors:** Agustín González-Fontes

**Affiliations:** 1 Department of Physiology, Anatomy and Cellular Biology, Pablo de Olavide University, Seville, Spain

This article is an extension and confirmation of findings on the degradation of the borate transporter BOR1 in *Arabidopsis thaliana *tobacco BY-2 cells. From elegant and conclusive techniques used in previous works by the Fujiwara and Matsuoka groups, the authors demonstrated that, under high B supply (3-5 mM), the BOR1 transporter in transformed tobacco BY-2 (BOR1-GFP) cells was also transported from the plasma membrane to the vacuole via endocytosis for degradation, this borate-dependent process being specific for BOR1-GFP. However, unlike in *Arabidopsis*, BOR1-GFP did not pass through the trans-Golgi network during degradation.

Interestingly, the authors showed that de novo synthesis of the borate-dependent degradation machinery of the borate transporter BOR1 is required for maintaining the degradation. However, it is noteworthy that the threshold B concentration for the onset of borate-dependent degradation in tobacco BY-2 cells was 3 mM, but in *Arabidopsis* root cells is only 0.1 mM B. The authors should give some additional explanation for this significant discrepancy, other than that the plant species have different high-boron sensitivity.

I have read this submission. I believe that I have an appropriate level of expertise to confirm that it is of an acceptable scientific standard.

# Peer review: Referee response for version 1

**Published:** 2013-10-28

**Authors:** Liwen Jiang

**Affiliations:** 1 School of Life Sciences, Chinese University of Hong Kong, Hong Kong, China

This is an interesting study on the degradative pathway and dynamics of BOR1-GFP in transgenic tobacco BY-2 cells. The conclusions are largely supported by the provided data. I welcome its publication as it is informative and useful for the study of endocytosis in plant cells.

Some minor suggestions:

1. Add scale bars to some of the Figures (e.g. Figures 1C, 3A, 4A, 7C, 8A).
2. Please indicate the molecular weight (kDa) of the detected proteins in Figures 1D, 3B and 5A.

I have read this submission. I believe that I have an appropriate level of expertise to confirm that it is of an acceptable scientific standard.

# Peer review: Referee response for version 1

**Published:** 2013-10-23

**Authors:** Tomomichi Fujita

**Affiliations:** 1 Faculty of Science, Hokkaido University, Sapporo, Japan

This paper demonstrates that in a heterologous plant system the Arabidopsis borate transporter BOR1 was polarly localized in tobacco BY-2 cells and was degraded in a borate-dependent manner as has been reported in Arabidopsis roots. Pharmacological analysis suggested both common and distinct routes for the BOR1 degradation between Arabidopsis roots and tobacco BY-2 cells. The authors also explored Tyr mutants of the BOR1-GFP in BY-2 cells and found an interesting, distinct response from Arabidopsis. Thus I think this article has enough quality of science to be approved in F1000Research. I have some comments and questions that should be considered and explained for the improvement of the article.

                            

1. Fig.1 A: I can see some green dots from BOR1-GFP in the cytosol. According to the rest of the results (e.g. Fig. 1C, 3A (-Borate), 4A (Bor1-GFP)) the fluorescent signal in the low concentration of borate media and in the log phase of the BY-2 line is mainly detected in plasma membranes but not in the cytosol; could you explain why you think this difference occured?
2. Fig.1 D: The authors fractionated the cell lysate to show that BOR1-GFP is targeted to the membrane fractions; it would be interesting to know whether either fraction between P10 and P100 contains more plasma membranes or more microsomes. It would also be helpful if the authors could add control experiments for each fraction using protein markers that are known to localize in plasma membranes, microsomes, or the soluble fraction.
3. p.4 left column: The authors used PIP-GFP, and then later SCAMP2-mRFP. It would be better to describe the origins (Arabidopsis or tobacco) of these in the Materials and Methods section.
4. Fig.2: The authors measured the cell volume of BY-2 cells. Please add the methods used and how many times the measurement was replicated (n=?).
5. Fig. 6A: In the picture, please add the note ‘ *Bor1-GFP’* as indicated in Fig. 6B and C.
6. Fig. 6C legend: Please check if the sentence ‘ *BOR1-GFP was transiently expressed in cells expressing SCAMP2-mRFP*…’ is not instead supposed to read ‘ *SCAMP2-mRFP was transiently expressed in cells expressing BOR1-GFP*.’
7. Fig. 6C: The authors examined the endocytic route for BOR1 degradation. Based on the observation in the merged picture, they concluded that since no overlapping of green and red dots was observed, co-localization of SCAMP2 and BOR1 was unlikely. However, I see only a few dots in the original picture of BOR1-GFP that they presented, and I wonder if this is why in the merged picture there were few yellow signals indicating an overlap of SCAMP2 and BOR1. The authors should show an appropriate BOR1-GFP image with more dots of BOR1-GFP, and the SCAMP2 image should be merged so that the colocalization can be observed. Additionally, even in the current merged picture, I can see at least one yellow dot at the periphery of the bottom-side plasma membrane; how is this dot explained?
8. p.6 right column, line-16: (Figure 6) should be (Figure 7).
9. Fig. 7C: The authors examined whether inhibition occurred at the point of endocytosis from the plasma membrane or at intermediate compartments. However, it is hard to distinguish if there are any intermediate compartments or not at the presented magnification/resolution of the images. Especially in the images for the TIBA treatment, as the relatively strong fluorescent signal was probably recognized in a surrounding region of the nucleus. Please show better images and explain if this is related to the intermediate compartments or something else.
10. p.11 left column, line 7 from below: ‘GFP8’ could be ‘GFP’?

I have read this submission. I believe that I have an appropriate level of expertise to confirm that it is of an acceptable scientific standard.
